# Parsonage–Turner syndrome following coronavirus disease 2019 immunization with ChAdOx1-S vaccine: a case report and review of the literature

**DOI:** 10.1186/s13256-021-03176-8

**Published:** 2021-12-13

**Authors:** Bruno Kusznir Vitturi, Marina Grandis, Sabrina Beltramini, Andrea Orsi, Angelo Schenone, Giancarlo Icardi, Paolo Durando

**Affiliations:** 1 Department of Health Sciences, University of Genoa, L.go R. Benzi, 10 (Building 3), 16122 Genoa, Italy; 2 DINOGMI, University of Genoa, Genoa, Italy; 3 Neurology Unit, IRCCS Ospedale Policlinico San Martino, Genoa, Italy; 4 Pharmacy Unit, IRCCS Ospedale Policlinico San Martino, Genoa, Italy; 5 Hygiene Unit, IRCCS Ospedale Policlinico San Martino, Genoa, Italy; 6 Occupational Medicine Unit, IRCCS Ospedale Policlinico San Martino, Genoa, Italy

**Keywords:** Acute brachial neuritis, Adverse reactions, COVID-19, COVID-19 vaccines, Neuralgic amyotrophy, Parsonage–Turner syndrome

## Abstract

**Background:**

Parsonage–Turner syndrome is an acute peripheral neuropathy that affects the upper brachial plexus region. Previously published reports demonstrate that the condition can be triggered by surgery, infection, autoimmune diseases, strenuous exercise, trauma, radiation, and vaccination. Parsonage–Turner syndrome has already been reported in three other patients who were vaccinated against coronavirus disease 2019.

**Case presentation:**

We report the case of a 51-year-old Caucasian man without comorbidities who received the first dose of the ChAdOx1-S recombinant vaccine (Vaxzevria, AstraZeneca, Oxford, UK) against coronavirus disease 2019 and was diagnosed with Parsonage–Turner syndrome. A few days after getting vaccinated, the patient reported a progressive increase in pain in the region of vaccine administration. One month later, the shoulder pain was followed by symptoms of hypoesthesia and muscle weakness on abduction and elevation of the left upper limb. Neurological examination revealed an atrophy of the proximal muscles of the left upper limb, accompanied by paresis of the left deltoid, biceps brachii, triceps brachii, and infraspinatus muscles. Electroneuromyography carried out 3 months after the onset of symptoms showed signs consistent with brachial plexus neuritis. The adverse reaction has been properly reported to the Italian Pharmacovigilance System (Italian Medicines Agency—*Agenzia Italiana del Farmaco*.

**Conclusion:**

The increased awareness of such association is essential for early identification and diagnosis and, thus, better clinical outcomes.

## Background

Parsonage–Turner syndrome, also known as idiopathic neuralgic amyotrophy or brachial neuritis, is an acute peripheral neuropathy that affects the upper brachial plexus region. The incidence rate is 1 in 1000 per year. The clinical phenotype usually includes excruciating pain in the proximal upper extremity followed by multifocal muscle weakness. Muscle atrophy and sensory symptoms may also occur [1]. Adhesive capsulitis, subacromial bursitis, facioscapulohumeral dystrophy, motor neuron disease, cervical radiculopathy, and entrapment neuropathies are generally the main differential diagnosis [2]. The etiology of Parsonage–Turner syndrome is still unclear, but it is thought to be an immune-mediated reaction against the brachial plexus nerve that occurs in genetically predisposed individuals. Previously published reports demonstrate that the condition can be triggered by surgery, infection, autoimmune diseases, strenuous exercise, trauma, radiation, and vaccination. Recently, brachial neuritis has been associated with severe acute respiratory syndrome coronavirus 2 (SARS-CoV-2) infection as well [3, 4]. Diagnosis is made based on the clinical history, physical examination, and electroneuromyography. Imaging examinations [computed tomography (CT) or magnetic resonance imaging (MRI)] can be useful to rule out other potential etiologies or when the electrophysiological study is inconclusive [5].

## Case presentation

We report the case of a 51-year-old Caucasian man who received the first dose of the ChAdOx1-S recombinant (Vaxzevria, AstraZeneca, Oxford, UK) coronavirus disease 2019 (COVID-19) vaccine and was diagnosed with Parsonage–Turner syndrome. The patient had no history of chronic diseases and did not use any continuous medications. His immunization schedule was complete, and he had never had any major vaccine reactions. He denied any recent trauma or infectious disease. The first clinical manifestations occurred shortly after vaccine administration. Initially, the patient presented fever, malaise, and asthenia and, 4 days later, there was a progressive increase in pain in the region of vaccine administration, which made him self-medicate with paracetamol, nonsteroidal anti-inflammatory drugs (NSAID), and pregabalin. One month later, the patient developed symptoms of hypoesthesia and muscle weakness on abduction and elevation of the left upper limb. Neurological examination revealed atrophy of the proximal muscles of the left upper limb, accompanied by paresis of the left deltoid, biceps brachii, triceps brachii, and infraspinatus muscles. There were no changes in superficial and deep sensation, and there were no motor deficits in other segments of the body. All deep tendon reflexes were normoactive and symmetrical. The patient was always lucid, oriented, and collaborative. No sensory deficits, fasciculations, or pathological upper motor neuron signs were seen. There were neither meningeal signs nor alterations in any cranial nerve.

Electroneuromyography (ENMG) carried out 3 months after the onset of symptoms showed signs consistent with brachial plexus neuritis. There was mild to moderate peripheral neurological damage with signs of reinnervation in the region of the deltoid, biceps brachii, triceps brachii, infraspinatus, extensor pollicis longus and brevis, and first interosseous muscles. A reduction in the amplitude of the left axillary nerve action potential was also observed. The clinical manifestations, the onset of symptoms soon after vaccine administration, and the absence of a past significant medical history, together with the physical examination findings and the typical alterations in electroneuromyography, allowed us to establish the diagnosis of Parsonage–Turner syndrome. The adverse reaction has been properly notified to the Italian Pharmacovigilance System (Italian Medicines Agency—*Agenzia Italiana del Farmaco* (AIFA), https://www.aifa.gov.it/en/web/guest/home). The patient was treated with NSAID, pregabalin, and physiotherapy. Five months after the initial presentation, he presented a partial recovery, persisting only with local muscle weakness.

## Discussion

Parsonage–Turner syndrome has already been reported in three other patients who were vaccinated against COVID-19 (Table 1) [6–8]. Patients aged between 35 and 51 years, and the time to onset of first symptoms ranged from 5 to 9 days after the first dose. In all patients, there were sensory symptoms and compatible pathological findings on electrophysiological study. The cases occurred after the administration of two different COVID-19 vaccines, suggesting that the syndrome can occur regardless of their mechanism of action.

**Table 1:** Clinical and demographic characteristics of Parsonage–Turner syndrome cases associated with COVID-19 vaccination published in the literature

Study	COVID-19 vaccine	Dose	Immunization schedule completed	Age	Sex	Time to onset of first symptoms	Motor symptoms	Sensory symptoms	Altered ENMG	Treatment	Recovery
Mahajan *et al*.	BNT162b (Comirnaty, Pfizer-BioNtech)	First	Yes	50 years old	M	7 days	Hand grip and wrist extension weakness	Pain	Yes	NSAID Corticosteroids	Partial recovery
Diaz-Segarra *et al*.	BNT162b (Comirnaty, Pfizer-BioNtech)	First	NA	35 years old	F	9 days	Arm weakness	Numbness and paresthesias	Yes	Corticosteroids	Partial recovery
Crespo Burillo *et al*.	ChAdOx1-S (Vaxzevria, AstraZeneca)	First	NA	38 years old	M	4 days	No	Pain	Yes	NSAID Corticosteroids Physiotherapy	Full recovery
Present case	ChAdOx1-S (Vaxzevria, AstraZeneca)	First	No	51 years old	M	4 days	Proximal muscle weakness of the left upper limb	Pain Hypoesthesia	Yes	NSAID Paracetamol Pregabalin Physiotherapy	Partial recovery

*NA* not available

There is no randomized controlled trial supporting an evidence-based approach to this syndrome, but it is generally accepted that current treatment may involve a combination of steroids, analgesics, and physiotherapy [9]. Due to the neuropathic nature of pain, the use of anticonvulsants can also be effective. Rehabilitation especially offers the possibility of recovery of motor function and should be encouraged early in association with pharmacological therapy, in order to obtain the best neurological outcomes in the medium and long term [9]. Most patients evolve with partial or full recovery at 3 years; however, it is noteworthy that more than 70% of patients may experience residual paresis [9]. Besides, among the clinical manifestations associated with a worse prognosis, the involvement of the phrenic nerve stands out.

At the time of the submission of this case report, there were more than 72 million doses of COVID-19 vaccines administered in Italy and three other cases of Parsonage–Turner syndrome reported as a vaccine adverse reaction to the Italian Medicines Agency—AIFA, one of which was associated with the ChAdOx1-S vaccine, one with the BNT162b vaccine, and another with the Ad26.COV.2 (Johnson & Johnson/Janssen) vaccine. All of them presented similar clinical manifestations; a favorable recovery was reported in one case (BNT162b vaccine), a partial recovery was outlined in another case (Ad26.COV.2 vaccine), and no information about the clinical evolution was available in the case that occurred following immunization with the ChAdOx1-S vaccine. Thus, the present report is the first case reported from Italy, with complete clinical information available, and echoes the three cited case reports already published in the literature. Even so, it is important to mention that it is not possible to determine a causal link between the administration of the vaccine and the neurological syndrome.

## Conclusion

Parsonage–Turner syndrome may be a rare adverse reaction to COVID-19 vaccines. It usually presents with intense pain in the proximal upper extremity followed by multifocal muscle weakness. This case report exemplifies that an increased awareness of such association is essential for an early identification and diagnosis and, thus, better clinical outcomes.

## Data Availability

Not applicable.

## Abbreviations

AIFA: *Agenzia Italiana del Farmaco*
ENMG: Electroneuromyography
MRI: Magnetic resonance imaging
CT: Computed tomography
NSAID: Nonsteroidal anti-inflammatory drugs
